# Real-world outcomes of managing autosomal-dominant polycystic kidney disease using a medical food as part of a nutrition and lifestyle program to improve renal and metabolic health

**DOI:** 10.3389/fnut.2025.1624639

**Published:** 2026-01-21

**Authors:** Emily Muensterman, Jacob A. Torres, Thomas Weimbs

**Affiliations:** Santa Barbara Nutrients, Inc., Santa Barbara, CA, United States

**Keywords:** ADPKD, ketogenic diet, beta-hydroxybutyrate, citrate, renal microcrystals, real-world evidence

## Abstract

**Background:**

Autosomal-dominant polycystic kidney disease (ADPKD) is a common hereditary form of chronic kidney disease with limited pharmacological treatment options. Emerging evidence suggests that metabolic interventions, including ketogenic dietary strategies and reducing lithogenic risk, may positively influence disease progression.

**Objectives:**

This study evaluated real-world outcomes from the Ren-Nu™ program, a remotely administered educational program for individuals with ADPKD that combines the use of a medical food with nutrition and lifestyle changes, including a very-low-carbohydrate ketogenic diet and reduction of renal lithogenic stressors. Ren-Nu™ was launched in 2021 as a collaboration between scientists and nutrition experts from the University of California Santa Barbara and Santa Barbara Nutrients, Inc, and dietitians from Kidney Nutrition Institute.

**Methods:**

Data from 103 ADPKD participants who completed the Ren-Nu™ program between 2021 and 2023 were analyzed in this longitudinal, baseline- controlled evaluation. The 3-month intervention included structured dietary education, regular dietitian and nutritionist support, and supplementation with KetoCitra^®^, a medical food providing beta-hydroxybutyrate (BHB), citrate, minerals, and alkali base. Primary outcomes included estimated glomerular filtration rate (eGFR), body mass index (BMI), anti-hypertensive medication usage, and self-reported symptom burden. Safety was assessed through routine metabolic biomarkers.

**Results:**

Participants demonstrated high adherence to nutritional ketosis, leading to a significant improvement in BMI. Renal function significantly improved, showing an eGFR increase of 6.3% (from 58.4 to 61.6 ml/min/1.73 m2; P < 0.001). There was a notable decrease in anti-hypertensive medication use and significant reductions in self-reported kidney pain and headaches. Safety markers, including lipid profiles, electrolytes, and acid-base balance, remained stable throughout the intervention.

**Conclusion:**

Use of KetoCitra^®^ supported by nutrition and lifestyle changes in the Ren-Nu™ program demonstrates feasibility, safety, and clinically meaningful improvements in metabolic health, renal function,  and quality

## Graphical Abstract

**Figure:**
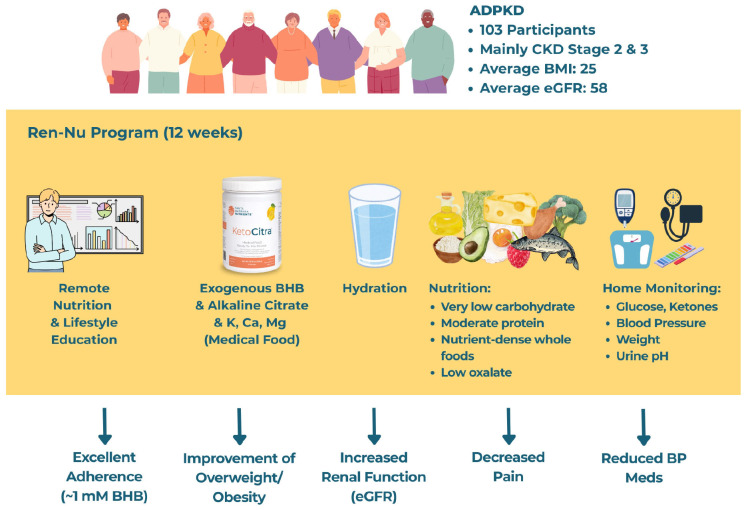

## Introduction

Autosomal-dominant polycystic kidney disease (ADPKD) is the most common inherited kidney disorder, affecting approximately 1 in 500–1,000 individuals worldwide (1). Characterized by progressive cyst development, ADPKD leads to renal enlargement, chronic kidney disease (CKD), and ultimately, end-stage renal disease (ESRD) in the majority of affected individuals. Despite the high disease burden, therapeutic options remain limited, with only one FDA-approved pharmacological agent, tolvaptan, available to slow disease progression. However, its use is constrained by adverse effects, high cost, and strict toxicity monitoring requirements (2, 3).

Recent studies suggest that factors influenced by nutrition and lifestyle contribute to ADPKD progression (4). This includes two primary causes of kidney injury: (1) lithogenic stress due to renal tubular microcrystal formation (such as calcium oxalate, phosphate, and uric acid), and (2) metabolic disease (persistent hyperglycemia and insulin resistance) (4). Preclinical studies show that mitigating these renal stressors profoundly slows, halts, or even partially reverses disease progression in rodent models of ADPKD. Mitigating the lithogenic stress by supplementation with alkaline citrate potently inhibits cyst formation and slows PKD progression (5, 6). Improving metabolic health by inducing nutritional ketosis using time-restricted feeding, periodic fasting, or a ketogenic diet not only slows PKD progression but can even lead to partial disease reversal in animal models (6–8). The beneficial effects of nutritional ketosis are largely mimicked by supplementing with the main ketone produced during ketosis, beta-hydroxybutyrate (BHB) (6–8). Further, combining supplementation with alkaline citrate and BHB showed synergistic beneficial effects on PKD progression in an animal model of PKD (6).

Recent human clinical studies support these interventions by showing potential benefits for individuals with ADPKD. A retrospective case series study of 131 individuals with ADPKD self-implementing ketogenic regimens for an average duration of 6 months suggested feasibility and safety, along with apparent improvements in renal function and ADPKD-associated health issues, such as hypertension and pain (9). A randomized controlled trial comparing a 3-month intervention with a ketogenic diet versus no diet change again showed feasibility and safety (10). Additionally, this trial showed statistically significant improvement of renal function based on creatinine and cystatin-C (10). A *post hoc* analysis also showed a statistically significant decrease in total kidney volume, a surrogate measure of ADPKD disease progression, in the ketogenic diet arm (11). Several other studies have also shown that ketogenic metabolic therapy is not only effective for the remission of type 2 diabetes but also improves diabetes-associated CKD (12, 13).

KetoCitra^®1^ is a medical food formulated for the daily dietary management of individuals with CKD, including ADPKD. It combines alkaline citrate and BHB with potassium, calcium, and magnesium. As a product that falls into the FDA-regulated medical food category, all ingredients in KetoCitra^®^ are generally recognized as safe (GRAS). To further support the use of KetoCitra^®^ in ADPKD management, the Ren-Nu™ program^2^ was developed. This 3-month, remotely delivered program integrates additional nutrition and lifestyle modifications aimed at reducing lithogenic stressors and enhancing metabolic health. The program emphasizes the safe implementation of a very-low-carbohydrate, ketogenic dietary pattern, while also aiming to reduce exposure to key lithogenic factors such as oxalate, phosphate, and uric acid. The foundational principles and key features of KetoCitra^®^ and the Ren-Nu™ program were initially published in 2022 (14). Since then, over 200 participants have completed the Ren-Nu™ program. Here, we report a retrospective program evaluation representing real-world outcomes and exploring the feasibility, safety, and effectiveness of this intervention.

## Materials and methods

### Study design

This retrospective evaluation of the Ren-Nu™ program is based on a collaboration between Santa Barbara Nutrients and Kidney Nutrition Institute, run from 2021 to 2024. This group nutrition intervention was led by specialized renal dietitians, implementing a low-oxalate, plant-heavy, ketogenic approach incorporating the BHB-citrate product, KetoCitra^®^, for individuals with PKD. Health data of Ren-Nu™ participants were routinely collected by the program dietitians and nutritionists at baseline (before starting nutritional changes), throughout, and at completion of the 3-month program.

### Setting, study population, and strategy

De-identified data were extracted from the Kidney Nutrition Institute’s clinical database between March 2021 and September 2023. Individuals with a confirmed diagnosis of ADPKD and an estimated glomerular filtration rate (eGFR) > 30 (mL/min/1.73 m^2^) qualified for enrollment in the program. Given the real-world nature of the intervention, this study did not employ randomization or a control group but compared outcomes within individuals from baseline to program completion.

### Intervention

The key features of the Ren-Nu™ program were previously published (14). Briefly, the intervention integrates the following nutrition and lifestyle features to support the unique needs of individuals with ADPKD:

1. Daily use of the medical food KetoCitra^®^, which provides 5.3 g of BHB, 3.5 g of citric acid, 600 mg of potassium, 300 mg of calcium, 250 mg of magnesium, and 51 mEq of alkaline base per recommended daily serving. KetoCitra^®^ is free from sodium and added sugars.
2. Adoption of a ketogenic lifestyle characterized by a low-carbohydrate, high-fat, moderate-protein diet, with the option for time-restricted eating. The goal is to achieve and maintain nutritional ketosis (blood BHB ≥ 0.5 mmol/L) while supporting glycemic control.
3. An omnivorous, plant-focused dietary pattern, emphasizing plant-based choices to promote alkalinity. Animal foods such as dairy, eggs, poultry, red meat, fish, and seafood are incorporated based on individual preferences.
4. Reduction of dietary renal stressors, particularly oxalate and inorganic phosphate additives, to help minimize lithogenic stress and prevent microcrystal formation in renal tubules.
5. Focus on whole and minimally processed foods to reduce the intake of ultra-processed products.
6. Fully remote program delivery to enhance accessibility and scalability.
7. Integration of digital tools and online platforms to support engagement, foster community, and facilitate remote monitoring and communication.
8. Education on the scientific rationale, practical skills, and mindset shifts needed to support long-term nutrition and lifestyle changes.
9. Self-monitoring of dietary intake and key health parameters, including body weight, blood pressure, blood glucose, blood ketones, and urine pH, to promote accountability and enable data-driven adjustments.
10. Ongoing supervision and individualized support from trained dietitians and nutritionists to ensure safety, address barriers, and optimize adherence.

This patient-centric, 100% virtual program is led by dietitians and nutritionists who educate and implement these nutrition and lifestyle changes over 4 months (1-month orientation, then a 3-month intervention). The curriculum includes weekly online group meetings with instruction modules and question and answer sessions, monitoring participants’ nutrient intake, symptoms, and progress each week, and conducting three individualized nutrition coaching sessions (at the beginning, the middle, and the end of the program). These nutrition and lifestyle approaches aim to provide a cost-effective and safe approach to long-term disease management.

The program utilized a variety of digital tools, including:

- A HIPAA-compliant online platform (Practice Better, Toronto, ON, Canada) to track outcome data, distribute materials, recipes, recorded videos, and facilitate discussion between participants, their peers, and the dietitian and nutritionist.
- The Cronometer smartphone app (Cronometer, Revelstoke, BC, Canada) was used by participants to track their daily macronutrient and micronutrient intake along with other health outcomes.
- The Keto-Mojo GK + Blood Glucose and Ketone Meter (Keto-Mojo, Napa, CA, USA), at-home blood pressure monitors, and body weight scales were used by participants to monitor their blood glucose and blood BHB levels, blood pressure, and body weight, respectively. Participants uploaded data to the Cronometer app for the dietitian and nutritionist’s review.

Key features of the medical food, KetoCitra^®^, are as follows. KetoCitra^®^ is a medical food specifically developed for the daily dietary management of individuals with mild to moderate ADPKD (CKD stages 1-3). KetoCitra^®^ is a ready-to-mix drink powder that, when mixed with water, provides individuals with BHB, citrate, alkaline base load, and inorganic electrolytes (potassium, calcium, and magnesium). The BHB delivered with KetoCitra^®^ is intended to support the dietary management of cellular metabolic abnormalities in ADPKD and support the state of ketosis. The citrate delivered with KetoCitra^®^ is intended to help normalize urinary citrate levels since hypocitraturia is common in ADPKD. The alkaline base load and inorganic electrolytes delivered with KetoCitra^®^ are intended to support the rebalancing of urine pH. This pH rebalancing effect addresses the known and abnormally low urine pH that is common among individuals with ADPKD, which further increases the risk of forming damaging renal microcrystals (15–17). Potassium delivered with KetoCitra^®^ is an essential nutrient for kidney function and has been demonstrated to be beneficial for supporting kidney health by reducing blood pressure and balancing sodium excretion (18, 19). The calcium and magnesium delivered with KetoCitra^®^ are intended to lessen the dietary absorption of oxalate and inorganic phosphate when taken with meals. Both oxalate and phosphate (20–23) may be damaging to kidneys due to renal microcrystal formation, and high intake has been shown to worsen PKD disease progression in animal models (5). If calcium and magnesium are taken together with foods, they will form insoluble complexes with oxalate and phosphate in the gastrointestinal tract, which reduce their absorption (24, 25).

### Data collection

Patient demographics and health and nutrition histories were collected during enrollment. During the program, participants completed self-reported surveys to measure dietary knowledge, progress, and potential adverse events. Dietitians and nutritionists worked with individual participants’ physicians to order labs at baseline and program completion and made informed decisions about treatment plans, such as managing any necessary medication adjustments, including antihypertensives.

The primary outcomes of this evaluation included the assessment of nutritional ketosis through serum BHB, metabolic health through body mass index (BMI) and body weight, renal function through eGFR (creatinine-based), medication deprescription focused on antihypertensive medication usage, self-reported ADPKD-associated symptom burden using a 10-point scale, and ketogenic diet safety markers, including lipids, electrolytes, and bicarbonate.

### Statistical analysis

Due to the real-world nature of the Ren-Nu™ program and the retrospective design of this study, data for all variables were not available for every patient. Therefore, the sample size (*n*) is specified for each variable. In total, this evaluation includes data points from 103 participants. The data analysis and graphing were conducted using Prism software (GraphPad Software, San Diego, CA, USA). Descriptive and clinical data were summarized using mean, standard deviation (SD), and percentage values. Paired *t* tests were used to compare baseline and completion data. Significance was set at *P* < 0.05.

## Results

### Participants

As of September 2023, 151 ADPKD patients completed the Ren-Nu™ program (Table 1). Data from 41 participants were unavailable for analysis, and 7 participants dropped out of the program. Reasons for non-completion included transitioning to a low-carbohydrate approach (*n* = 6) and unresolved health issues (*n* = 1). Data from 103 participants were available for analysis.

**TABLE 1:** Ren-Nu participants demographics.

Ren-Nu participants	
Participants completed the program as of September 2023	151
Participants whose data were not available for analysis	41
Did not complete the program	7
Reason for not completing	Moved to low carb approach: 6 Health issue: 1
Participants included in the analysis	103

SD, standard deviation.

The demographics, anthropometrics, and baseline data of the participants are shown in Table 2. Out of 103 participants, 68.9% (*n* = 71) were female, and the median age at baseline was 50 years (*n* = 84). The mean starting BMI was 25.0 ± 4.4 (*n* = 35), body weight was 170.8 ± 49.8 lbs (*n* = 45), systolic blood pressure was 124.4 ± 12.1 mmHg (*n* = 35), and diastolic blood pressure was 78.5 ± 8.0 mmHg. Mean creatinine levels were 1.30 ± 0.41 mg/dl (*n* = 85), and mean eGFR was 58.4 ± 21.07 ml/min/1.73 m^2^ (*n* = 77). The CKD stage distribution was as follows (Table 3): CKD 1 (GFR ≥ 90) 9.0%, CKD 2 (GFR 60–89) 32.5%, CKD 3 (GFR 30–59) 53.3%, and CKD 4 (GFR 15–29) 5.2%.

**TABLE 2:** Demographics, anthropometrics, and baseline data.

Demographics, anthropometrics, and baseline data (mean, SD)	
Age (years)	50 ± 10.7, *n* = 84
Gender	Male: *n* = 32 (31.1%) Female: *n* = 71 (68.9%)
BMI	25.0 ± 4.4, *n* = 35
Body weight (lbs)	170.8 ± 49.8, *n* = 45
Blood pressure – systolic (mmHg)	124.4 ± 12.2, *n* = 35
Blood pressure – diastolic (mmHg)	78.54 ± 8.09, *n* = 35
Creatinine (mg/dl)	1.30 ± 0.41, *n* = 85
eGFR (ml/min/1.73 m^2^)	58.40 ± 21.07, *n* = 77

SD, standard deviation.

**TABLE 3:** Chronic kidney disease (CKD) stage demographics.

CKD stage	
CKD 1 (GFR = 90 or higher)	9.00%
CKD 2 (GFR = 60–89)	32.50%
CKD 3 (GFR = 30–59)	53.30%
CKD 4 (GFR = 15–29)	5.20%

### Adherence and ketosis

**FIGURE 1:**
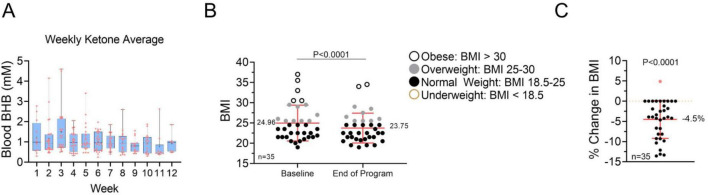
Ketosis induction and change in body mass index (BMI). **(A)** Weekly blood β-hydroxybutyrate (BHB) levels were measured for each participant to assess nutritional ketosis. Individual values within quartiles are shown. The + sign denotes the mean of each group. **(B)** The BMI of participants was measured at baseline and the end of the program. Individual values are depicted, and the means and standard deviations are indicated. A paired *t*-test was used to compare the change between groups. **(C)** Percent change in BMI of individuals within the study. A one-sample *t*-test was used to analyze the effect of the intervention.

During the fat-adaption phase (lasting the first 4–8 weeks of the intervention), the target was to maintain ketone levels between 1.0 and 2.0 mmol/L. Upon achieving fat adaptation, generally occurring by week 8, the goal shifted to sustaining ketone levels of 0.5 mmol/L or higher for the remainder of the program. Fat adaptation was defined by consistent carbohydrate intake and sustained ketosis, verified by program dietitians and nutritionists using data from Cronometer (diet tracking) and Keto-Mojo (ketone monitoring). Common indicators of fat adaptation included reduced cravings, stable energy levels, improved sleep, enhanced focus, decreased appetite, satiety between meals, improved endurance performance, and more stable blood glucose levels. Participants maintained consistent nutritional ketosis throughout the program, as demonstrated by serum BHB measurements (Figure 1A), indicating adherence to the intervention.

### Weight loss and BMI reduction

The average BMI (*n* = 35) significantly decreased across participants, reflecting successful fat loss and improved metabolic status (Figures 1B, C). The average percent change in BMI was −4.5%. Many participants transitioned from the classification of obese to overweight, or from overweight to normal weight. No participant became underweight.

### Renal function

**FIGURE 2:**
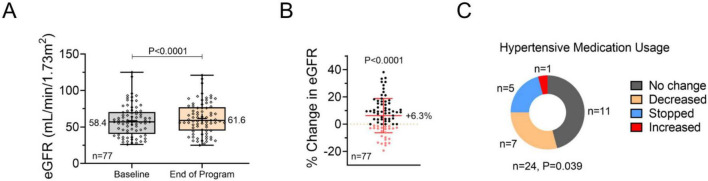
Renal function and change in medication usage. **(A)** Estimated glomerular filtration rate (eGFR, creatinine-based) of individuals at baseline and the end of the program. Individual values are shown within quartiles along with the mean of each group. A paired *t*-test was used to analyze the effect of the intervention. **(B)** The percent change in the eGFR of individuals within the study. Individual values and the standard deviation are shown with the mean value. A one-sample *t*-test was used to analyze the effect of the intervention. **(C)** Change in antihypertensive medication usage among participants from baseline to the end of the program. A Chi-square goodness of fit test was used to analyze the effect of the intervention.

The creatinine-based eGFR (*n* = 77) improved from the baseline average of 58.4 to the end-of-program average of 61.6, which was statistically significant (*P* < 0.0001). This reflects an average increase of +6.3% from baseline to program completion (Figures 2A, B).

### Medication usage

A significant proportion of participants reduced or discontinued anti-hypertensive medications during Ren-Nu™ (Figure 2C). At the start of the program, 24 participants reported using anti-hypertensive medication. By the end of the program, 11 participants reported no change, 7 decreased their dosage, 5 discontinued their medication, and 1 increased their dosage (*P* = 0.039).

### Symptom burden

**FIGURE 3:**
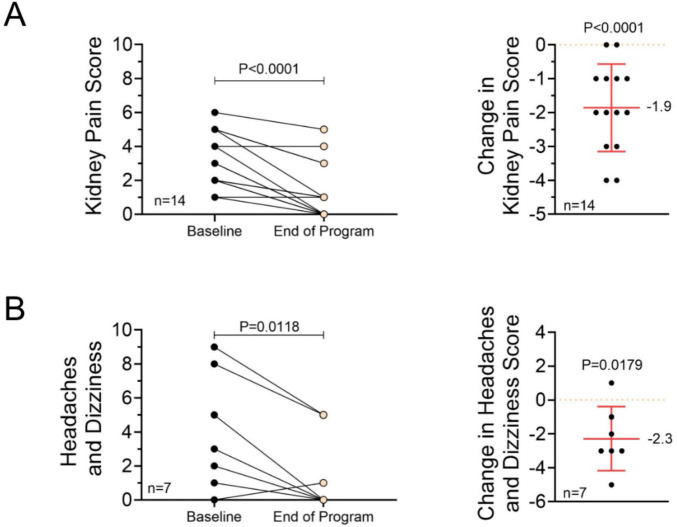
Changes in symptom burden. **(A,B)** Twenty-nine respondents completed the symptom questionnaire. Participants who reported a score of 0 at baseline and the end of the program were removed from the analysis. **(A)** Change in self-reported kidney pain scores from baseline to the end of the program, presented as raw scores on a 1–10 scale and as absolute change from baseline. A paired *t*-test and a one-sample *t*-test were used to evaluate the effect of the intervention. **(B)** Change in self-reported headache and dizziness scores from baseline to program completion, shown as raw scores on a 1–10 scale and as absolute changes from baseline. A paired *t*-test and a one-sample *t*-test were used to analyze the effect of the intervention.

Participants reported clinically meaningful reductions in kidney pain and headache or dizziness frequency/severity (Figure 3). At baseline and program completion, participants completed self-reported surveys on pain and other symptoms using 10-point scales. Those who reported no symptoms (score of zero at baseline and end of program) were excluded from the final analyses. Among participants reporting kidney pain (*n* = 27), 52% (*n* = 14) experienced a reduction in pain from baseline to the end of the program, while 48% (*n* = 13) reported no change. Notably, no participants reported an increase in kidney pain. This change in kidney pain (*n* = 14) was statistically significant (*P* < 0.0001). For headaches or dizziness (*n* = 27), 22% (*n* = 6) of participants reported a decrease, 74% (*n* = 20) reported no change, and 4% (*n* = 1) reported an increase. A change in headaches or dizziness (*n* = 7) was also statistically significant (*P* = 0.018). No significant changes were observed in other self-reported symptoms, including fullness, fatigue, energy level, sleep quality, stiffness, stress management, swelling, nausea or vomiting, constipation, or diarrhea.

### Safety

There were no significant adverse changes in lipid profiles or key metabolic safety markers. Total cholesterol (*n* = 26) remained stable, with a baseline average of 193.69 ± 32.54 mg/dl and an end-of-program average of 198.88 ± 40.38 mg/dl (*P* = 0.510). HDL cholesterol (*n* = 24) showed no significant change (60.92 ± 18.67 to 61.33 ± 18.94 mg/dl, *P* = 0.854), nor did LDL cholesterol (*n* = 23) (111.26 ± 25.46 to 117.22 ± 30.81 mg/dl, *P* = 0.402) or triglycerides (*n* = 16) (107.56 ± 68.84 to 91.81 ± 37.53 mg/dl, *P* = 0.263). Similarly, potassium (*n* = 44) remained stable (4.34 ± 0.43 to 4.40 ± 0.38 mmol/L, *P* = 0.207), as did phosphorus (*n* = 23) (3.48 ± 0.80 to 3.67 ± 0.77 mg/dl, *P* = 0.279) and bicarbonate (*n* = 45) (24.33 ± 3.19 to 24.29 ± 3.17 mmol/L, *P* = 0.927). Altogether, these results support the safety of the intervention.

## Discussion

This real-world evaluation demonstrates that the medical food KetoCitra^®^, in conjunction with Ren-Nu™, a comprehensive ketogenic nutrition and lifestyle program, tailored specifically for individuals with ADPKD, is both feasible and safe. Moreover, it is associated with significant clinical benefits. Observed improvements in renal function, body weight, blood pressure, and self-reported symptom burden support the potential of targeting underlying metabolic dysfunction and lithogenic risk in modifying the trajectory of a disease that has traditionally been viewed as relentlessly progressive.

These findings are consistent with studies indicating that ketosis and BHB can affect numerous processes that are known to be mechanistically involved in ADPKD progression, including anti-inflammatory effects and inhibition of the NLRP3 inflammasome, inhibition of the mTOR pathway, improved mitochondrial function, reduction of oxidative stress, and enhancement of autophagy. Alkalization and citrate supplementation, meanwhile, are expected to prevent renal microcrystal formation, a known inducer of cystogenesis. We suggest that the observed clinical benefits of KetoCitra^®^ and the Ren-Nu™ Program represent disease-modifying effects of a combination of mechanisms that may be additive or synergistic.

The observed eGFR improvement is particularly notable because renal function decline is commonly thought to be progressive in ADPKD. The average improvement of +6.3% in eGFR in the Ren-Nu™ program is consistent with a similar improvement found in a randomized-controlled clinical trial investigating a 3-month ketogenic dietary intervention. This study reported a creatinine-based eGFR increase of 5.5% and a cystatin-C-based eGFR increase of 13.9% (10). The finding that eGFR increased based on both markers of renal function suggests that the observed effect is unlikely to be attributable to changes in protein or creatine intake, or changes in muscle mass, as cystatin-C is independent of these factors. Additionally, several clinical studies investigating ketogenic diets in individuals with diabetic CKD have consistently demonstrated eGFR improvements, even in long-term studies up to 19 years, as summarized in recent review articles (12, 13). These findings suggest that improved renal function observed with ketogenic metabolic therapy is not simply due to a short-term effect on glomerular filtration.

### Limitations

This analysis has several limitations. First, the lack of a formal control group limits the ability to definitively attribute observed improvements to the intervention alone, as external influences cannot be fully excluded. However, this study employed a baseline-controlled design in which each participant served as their own control, a method that can offer advantages over comparisons between non-equivalent groups. Notably, spontaneous remission is not a recognized feature of ADPKD under current standard care (26–28). Therefore, improvements in clinical markers such as eGFR are unlikely to reflect natural disease fluctuation. In this context, the baseline-controlled design provides meaningful insight, particularly when findings include signs of remission (29). It is highly improbable that the majority of participants in this analysis would have experienced such improvements if they had not participated in the program. Second, participants self-selected into the Ren-Nu™ program, which may introduce selection bias. Individuals who choose to engage in a structured nutrition and lifestyle intervention may differ from the broader ADPKD population in terms of motivation, health literacy, or willingness to adopt behavior change. However, it is important to note that most participants reported longstanding symptom progression before enrollment, and declining health was a primary driver for seeking out Ren-Nu™. This suggests that while participants may have been more proactive, their baseline disease trajectory was consistent with the expected progression of ADPKD. Third, the Ren-Nu™ program is a multimodal intervention, which limits the ability to isolate the effects of individual components such as dietary education and support, community engagement, adherence to a ketogenic diet, reduction in lithogenic risk factors, and the use of the medical food KetoCitra^®^. However, this integrated approach was deliberate. The program was designed to combine multiple evidence-informed strategies that are each plausibly beneficial for individuals with ADPKD to provide additive or even synergistic effects. Fourth, limited or no information was available on potential mitigating treatments for PKD-related symptoms that may have occurred outside of Ren-Nu™. Therefore, it is possible that reported symptom improvements in some participants (kidney pain and headaches) may have been due to such external interventions. However, given that all participants were long-term sufferers from ADPKD it is unlikely that many participants would have initiated new treatments coincident with participating in Ren-Nu™. Fifth, while this study demonstrated the real-world feasibility of Ren-Nu™, it did not account for widespread socioeconomic factors that may impact adherence and long-term sustainability. Variables such as food accessibility, community support, cultural dietary norms, and the level of awareness among healthcare providers play a significant role in the successful adoption of lifestyle interventions. For example, individuals in lower-income communities or those with limited nutrition education may face greater obstacles in implementing and maintaining the dietary recommendations of the program (30). Lastly, data collection was heterogeneous, a common characteristic of real-world evaluations. Many measurements, including blood pressure and body weight, were self-reported using at-home monitoring devices. While this approach enhances accessibility and feasibility, it also introduces potential inconsistencies in measurement accuracy due to variations in device calibration, user technique, and adherence to measurement protocols.

### Future directions

A larger, prospective, controlled clinical trial with 1-year follow-up is currently underway at Juntendo University (Tokyo, Japan; registration UMIN000052907) to examine the long-term effects of the medical food KetoCitra^®^ in the context of the Ren-Nu™ program’s nutrition and lifestyle changes in ADPKD. Several additional trials are in planning.

## Conclusion

This evaluation of the Ren-Nu™ program suggests that educating individuals with ADPKD on the use of the medical food KetoCitra^®^, along with very-low-carbohydrate ketogenic nutrition and lifestyle changes, and measured to reduce the lithogenic risk, offers potential benefits for metabolic health, renal function, and symptom control. Participants could largely adhere to the program, and no significant safety concerns were observed. These encouraging real-world results support the continued investigation of these interventions as part of comprehensive ADPKD care.

## Data Availability

The raw data supporting the conclusions of this article will be made available by the authors, without undue reservation.

## Footnotes

1
https://santabarbaranutrients.com/

2
https://ren-nu.org/
